# Abiotic and Biotic Stress of the Crops and Horticultural Plants

**DOI:** 10.3390/plants14131910

**Published:** 2025-06-21

**Authors:** Pingfang Yang

**Affiliations:** School of Life Sciences, Hubei University, Wuhan 430062, China; yangpf@hubu.edu.cn; Tel.: +86-186-0715-0475

As sessile organisms, plants are continuously exposed to ever-changing environments during their life cycle. Some of these changes might be threats to plants’ normal growth and development and hence are defined as stresses, including biotic and abiotic stresses. To survive these stresses, plants have developed sophisticated and fine-tuned responsive mechanisms over the long period of their evolution. Undoubtedly, it is important to explore these mechanisms. Crops and horticultural plants are the two major groups of plants that are highly associated with the everyday life of human beings. Exploring the underlying mechanisms will not only provide new insights into plant stress responses but also facilitate the breeding of new crops or horticultural plants with high resistance to stress.

Drought is among the most well-known severe threats to crop productivity and quality all over the world, where a single drought can lead to a ~10% loss in cereal production. This is also the case in horticultural plant production. Sweet cherry is usually subjected to a summer water deficit in Chile, which could lead to very obvious growth defects because of the constraint on gas exchange [1]. In plants, evolved regulatory mechanisms to enhance resistance exist at both genetic and epigenetic levels. Recently, more and more studies have been conducted focusing on regulations that occur at the epigenetic level, which include three main types: DNA methylation, histone modifications, and RNA-mediated gene silencing [2]. Among them, DNA methylation includes 5-methylcytosine (5mC) and N6-methyladenosine (6mA), which are detected as epigenetic markers in eukaryotes. In plants, 5mC is predominantly detected in CG, CHG, and CHH sequence contexts [2]. Numerous studies have shown that DNA methylation plays an important role in plants in response to environmental factors through regulating gene expression. To update the latest understanding of the roles of DNA methylation in plant drought response, Rao et al. [2] reviewed most of the studies on this aspect in this Special Issue. Similar to crops, economic horticultural plants are also severely threatened by drought. Blueberry is an important fruit-bearing shrub. Its roots are shallow and hairless, which is not conducive to water and nutrient uptake. Therefore, it is important to clarify the mechanism underlying blueberry root drought tolerance. A yeast expression library comprising blueberry genes associated with root responses to drought stress was established, which enabled the identification of numerous genes and metabolites potentially related to drought tolerance [3]. Characterization of the genes that play roles in drought response is one of the major focuses. Through genome-wide analysis of the Laccase gene family in *Fragaria vesca*, *FvLAC51* was identified to play positive roles, while *FvLAC24* and *FvLAC32* play negative roles in both drought and salinity stresses [4]. Crops’ tolerance to drought is also affected by microbes. It was found that strains of the genus *Pantoea* could generate high ACC deaminase activity to enhance coffee plant resilience to drought [5].

Salinity is another frequently occurring abiotic stress, especially in semi-arid regions, which severely affects the growth and productivity of crops and horticultural plants. In a somewhat similar vein to drought, salinity could also lead to osmotic stress in cells, to which the response is largely mediated by a phytohormone, ABA. Because of this, there are common pathways through which crops could increase their tolerance. This is supported by the fact that some members of the *FvLAC* gene family could play either positive or negative roles in *Fragaria vesca* [4]. The primary response of plants to salinity is to increase the concentration of osmolytes, such as proline. In fact, the application of exogenous proline enhanced the growth of Nasturtium (*Tropaeolum majus* L.) under salinity stress [6].

Temperature is one of the major factors that determine the distribution of plants, and hence the cultivation area of crops. Global climate change has led to the frequent occurrence of extreme temperatures, including cold and heat. Cold can cause obvious yield and quality decreases in crops, especially in northern areas or over the winter season. Blueberry cultivated in Liaodong of China is facing such a threat [7]. A comprehensive transcriptomic and proteomic analysis identified some calcium-related genes (*CDPK*s and *CML*s), glutathione proteins, and TFs (NAC, WRKY, and ERF) that might be involved in blueberry cold tolerance [7]. Hence, these genes could be used as markers for blueberry breeding to improve cold tolerance. Low temperature is also a challenge for rice seed germination in the early season of double cropping. To assure unified germination and seedling growth, Zhang et al. applied a phytohormone priming method and found that GA_3_ is the most effective phytohormone [8]. Seed germination is a physiological process that is regulated by many endogenous and exogenous factors. As for the endogenous factors, besides phytohormones, physical barriers are another important factor affecting seed germination. Although physiological dormancy has been well characterized, physical dormancy has not been widely studied. Undoubtedly, it is also very important to elucidate the molecular mechanism underlying the formation of physical dormancy [9].

In addition to abiotic stresses, biotic stresses also seriously threaten the productivity of all kinds of crops. Sometimes, the microbes that exist in the grains or edible tissues of crops can negatively affect the quality and safety of processed foods derived from the crops. For example, the processing of malting barley is often affected by *Fusarium* spp. It has been shown that pulsed electric field (PEF) treatment could up-regulate the expression of some pathogen-related genes, which helps it to inactivate the pathogen and mitigate the effects of its infection [10]. It seems that PEF could be applied as an effective method to improve the quality of barley malt under abiotic stress. Interestingly, watered conditions had a great effect on sweet cherry’s resistance to *Pseudomonas syringae pv. Syringae* [1]. These examples indicate that there are mutual effects between biotic and abiotic stresses.

The leaf is the primary tissue where photosynthesis happens. Its photosynthesis efficiency largely determines the yield and quality of grains or fruits. The content of pigments, including chlorophylls and carotenoids, is very important for photosynthesis and hence could determine the quality of fruit in grape [11]. However, each leaf has its own life span and will be subject to senescence. If senescence occurs in a premature leaf, the yield and quality will both be negatively affected. Undoubtedly, it is necessary to explore the mechanism of senescence. It was found that leaf senescence might be regulated by WRKY transcription factors, specifically MsWRKY5, MsWRKY66, MsWRKY92, and MsWRKY141, in alfalfa [12].

The purpose of studying the mechanism underlying crop responses to stress is not only to obtain new insights but also to help crop breeding efforts. Based on this, it is very important to dig out the key genes that play roles in crop stress resistance, especially those genes that play dual roles in both biotic and abiotic stresses. It was found that calcium-dependent protein kinases (CPKs) are involved in drought and salt stress responses, and pathogen defense in rice and kiwifruit [13]. The B-box (BBX) protein has also been shown to have an impact on responses to both biotic and abiotic stresses in *Trichosanthes kirilowii* [14]. To breed highly resistant cultivars, it is necessary to develop highly efficient transformation techniques that can be widely applied in crops and horticultural plants. Accordingly, we also accepted one study in which a highly efficient Agrobacterium rhizogenes-mediated hairy root transformation method was developed for *Idesia polycarpa* [15]. With more knowledge and candidate genes, the breeding of crops and horticultural plants with high resistance to different stresses will see substantial benefits, which will help to ensure the security of food production around the world.
